# Development and evaluation of three mortality prediction indices for cold-stunned Kemp's ridley sea turtles (*Lepidochelys kempii*)

**DOI:** 10.1093/conphys/cot003

**Published:** 2013-04-19

**Authors:** N. I. Stacy, C. J. Innis, J. A. Hernandez

**Affiliations:** 1 University of Florida, College of Veterinary Medicine, Large Animal Clinical Sciences, 2015 SW 16th Avenue, Gainesville, FL 32610, USA; 2 New England Aquarium, 1 Central Wharf, Boston, MA 02110, USA

**Keywords:** Blood gas, chemistry, cold-stunning, Kemp's ridley sea turtle, mortality, prognosis

## Abstract

Kemp's ridley sea turtle is an endangered species found in the Gulf of Mexico and along the east coast of the USA. Cold-stunned Kemp's ridley turtles are often found stranded on beaches of Massachusetts and New York in November and December each year. When found alive, turtles are transported to rehabilitation centres for evaluation and treatment. Blood gas and chemistry analytes of major clinical relevance in sea turtles were selected to develop mortality prediction indices (MPI)s. Testing the diagnostic performance of various combinations of blood gas and chemistry analytes by receiver operating characteristics (ROC) analysis resulted in the development of three mortality prediction indices. The sensitivity and specificity of the best performing MPI (based on three blood analytes: pH, pO_2_, and potassium) was 88 and 80%, respectively. Using ROC analysis, the area under the curve = 0.896 (95% confidence interval = 0.83–0.94). The use of validated MPIs based on four blood analytes (pH, pCO_2_, pO_2_, and potassium) could be useful for better diagnosis, treatment, and prognosis of cold-stunned sea turtles when admitted to rehabilitation facilities.

## Abstract (precis)

Cold-stunned sea turtles can be affected by severe physiologic derangements, which if not treated appropriately, can lead to mortality. Clinically useful blood gas and chemistry analytes were selected to develop mortality prediction indices to improve diagnosis, medical treatment and prognosis during rehabilitation of cold-stunned sea turtles.

## Introduction

Kemp's ridley sea turtle is an endangered species found in the Gulf of Mexico and along the east coast of the USA (Marquez-Millan *et al.*, 2005). Juvenile Kemp's ridley turtles frequently forage in waters off the coast of New England in summer months. In late autumn, turtles that do not migrate away from this area may become cold-stunned as local water temperatures drop below 10°C (Burke *et al.*, 1991; Still *et al.*, 2005). Cold-stunned Kemp's ridley turtles are often found stranded on beaches of Massachusetts and New York in November and December each year (Morreale *et al.*, 1992; Morreale and Standora, 2005). When found alive, turtles are transported to rehabilitation centres for evaluation and treatment. Details of common physiological derangements, pathological conditions, and medical management of cold-stunned Kemp's ridley turtles have been described (Sadove *et al.*, 1998; Wyneken *et al.*, 2006; Innis *et al.*, 2007, 2009a, 2009b; Keller *et al.*, 2012). Physiological derangements in these studies included metabolic and respiratory acidosis, electrolyte imbalances, dehydration, and impaired renal function. Turtles affected by severe metabolic or respiratory derangements (e.g. acidosis, hyperkalemia) have a poor prognosis (Keller *et al.*, 2012).

In human and veterinary medicine, various illness scoring systems have been developed for diagnosis, treatment, and prognosis of disease in patients (Knaus *et al.*, 1985; Koterba and House, 1996; Lofstedt *et al.*, 1999; King *et al.*, 2001). A system for classifying cold-stunned sea turtles into one of four severity categories based on physical examination and behavioural observations was proposed by Sadove *et al.* 1998. However, the description provided only qualitative language for the prognosis of turtles in each category (e.g. class III turtles had the ‘poorest recovery rate’), and data were not provided to document whether the classification system performed well as a predictive tool. The use of a validated mortality prediction index (MPI) could be useful for better diagnosis, treatment, and prognosis of cold-stunned sea turtles when admitted to rehabilitation facilities. Here we describe the development of three indices to predict survival probability of cold-stunned Kemp's ridley sea turtles, evaluation of the performance of the indices when applied to a population of stranded turtles with known outcome, and modifications to improve the indices for proposed future use.

## Materials and methods

### Study population

One hundred and fifty-six juvenile stranded, cold-stunned Kemp's ridley sea turtles that were admitted alive to the New England Aquarium (NEAQ) during October 2010 to December 2011 were initially considered for inclusion. Criteria for inclusion in the study included cold-stunning as previously described for Kemp's ridley sea turtles admitted to the NEAQ (Innis *et al.*, 2007, 2009b; Keller *et al.*, 2012) and availability of a complete set of analytes from admission blood data. Of the turtles that died after admission, only turtles that died naturally within the first 7 days after admission were included. Twelve turtles that died later during rehabilitation (i.e. ≥8 days) were excluded because they died from conditions to which their admission blood data were no longer relevant. One turtle that was euthanized was excluded so that results were not biased by this subjective clinical decision. Thus, the final enrolment was 143 turtles. Out of these 143 turtles, 25 (17.5%) died naturally within the first 7 days after admission (mean = 2 days).

### Data collection

Data were reviewed retrospectively from all cold-stunned Kemp's ridley sea turtles admitted to the NEAQ during 2010 and 2011 that met inclusion criteria as described above. Admission blood data were defined as results from analysis of a venous blood sample collected on the day of admission to the NEAQ, prior to administration of any treatments (e.g. parenteral fluids). Venous blood samples had been collected anaerobically from the jugular vein into a heparinized 1 or 3 ml syringe and analysed immediately by use of a clinical point-of-care analyser (Critical Care Express; NOVA Biomedical, Waltham, MA, USA).

For each sea turtle included in this study, the following admission data were retrieved from medical records: blood pH; partial pressures of carbon dioxide and oxygen (pCO_2_ and pO_2_); concentrations of sodium, potassium, chloride, ionized calcium, and glucose; osmolality, and anion gap. The osmolality and anion gap data were calculated by the analyser. Values for pH, pCO_2_, and pO_2_ were corrected for the patient's body temperature (measured via a temperature probe inserted ∼10 cm into the cloaca), and ionized calcium was corrected for pH using published equations (Innis *et al.*, 2007). Bicarbonate concentration was calculated using the Henderson–Hasselbalch equation, temperature-corrected pH and pCO_2_, and values of αCO_2_ and p*K* calculated for each patient using previously described species-specific equations for Kemp's ridley turtles (Stabenau and Heming, 1993).

### Development of initial mortality prediction index scoring systems

In order to evaluate and quantify the severity of biochemical derangements in individual turtles objectively, three MPI scoring systems (MPI1, MPI2, and MPI3) were developed (Tables 1, 2, and 3). Blood analytes of interest and score values for each analyte (low and high abnormal ranges = 1–4, or 2–8) were selected based on relevance in the clinical assessment of cold-stunned Kemp's ridley turtles (Innis *et al.*, 2009b; Keller *et al.*, 2012), as well as clinical experience of two investigators (C.J.I. and N.I.S.). Three MPI scoring systems were developed using different combinations of blood analytes, with anticipation that at least one of the three would be more accurate in predicting mortality in sea turtles within 7 days after admission. Turtles with higher scores were categorized as physiologically deranged to a degree that could result in death, and turtles that received lower scores were categorized as physiologically stable and likely to survive. Categorization of each turtle was then compared to the known outcome for that individual.

**Table 1:** Mortality prediction index 1 (MPI1) scoring system for assessment of cold-stunned Kemp's ridley sea turtles

MPI1	High abnormal range				Low abnormal range			
Number of points to assign	8	6	4	2	2	4	6	8
pH	≥8.0	7.90–7.99	7.80–7.89	7.70–7.79	7.46–7.50	7.41–7.45	7.35–7.40	≤7.34
pCO_2_ (mmHg)	≥50	46–49	40–45	35–39				
Sodium (mmol/l)	≥180	170–179	165–169	160–164			136–140	≤135
Potassium (mmol/l)	≥6.0	5.5–5.9	5.0–5.4	4.5–4.9	2.5–2.9	2.0–2.4	1.5–2.0	<1.5

Abbreviation: pCO_2_, partial pressure of carbon dioxide.

**Table 2:** Mortality prediction index 2 (MPI2) scoring system for assessment of cold-stunned Kemp's ridley sea turtles

MPI2	High abnormal range				Low abnormal range			
Number of points to assign	4	3	2	1	1	2	3	4
Glucose (mmol/l)	≥15	12.5–14.9	10–12.4	7.5–9.9	2.3–2.5	1.8–2.2	1.3–1.7	1.2
pH	>7.9	7.7–7.9				7.41–7.50	7.35–7.40	≤7.34
pCO_2_ (mmHg)	≥50	40–49	35–39					
pO_2_ (mmHg)						41–50	31–40	≤30
Sodium (mmol/l)	≥165	160–164						
Potassium (mmol/l)	≥5.5	5.0–5.4	4.5–4.9		2.5–2.9	2.0–2.4	1.5–1.9	<1.5
Ionized calcium (mmol/l)	≥1.2	1.1–1.19					0.61–0.65	≤0.6

Abbreviations: pCO_2_, partial pressure of carbon dioxide; and pO_2_, partial pressure of oxygen.

**Table 3:** Mortality prediction index 3 (MPI3) scoring system for assessment of cold-stunned Kemp's ridley sea turtles

MPI3	High abnormal range				Low abnormal range			
Number of points to assign	8	6	4	2	2	4	6	8
Glucose (mmol/l)	≥15	12.5–14.9	10–12.4	7.5–9.9	2.3–2.5	1.8–2.2	1.3–1.7	1.2
pH	≥8.0	7.90–7.99	7.80–7.89	7.70–7.79	7.46–7.50	7.41–7.45	7.35–7.40	≤7.34
pCO_2_ (mmHg)	≥50	40–49	35–39					
pO_2_ (mmHg)					41–50	36–40	31–35	≤30
Sodium (mmol/l)	≥165	160–164						
Potassium (mmol/l)	≥5.5	5.0–5.4	4.5–4.9		2.5–2.9	2.0–2.4	1.5–1.9	<1.5
Ionized calcium (mmol/l)	≥1.2	1.1–1.19					0.61–0.65	≤0.6
Anion gap (mmol/l)	≥40	35–39	30–34	25–29				
Osmolality (mOsm/kg)	≥450	400–449	350–399					
Bicarbonate (mmol/l)	≥45	40–44	35–39					<5
Chloride (mmol/l)	≥141	136–140	131–135	125–130		105–110	104–100	<99

Abbreviations: pCO_2_, partial pressure of carbon dioxide; and pO_2_, partial pressure of oxygen.

### Data analysis

Receiver operating characteristic (ROC) analysis was used to assess the diagnostic performance of each MPI scoring system (Greiner *et al.*, 2000; Giguere *et al.*, 2003). The ROC analysis produces a plot that is used to estimate the area under a ROC curve, which is a summary statistic of diagnostic accuracy. A perfect test [i.e. sensitivity (SE) = 100% and specificity (SP) = 100%] will produce an area under the curve (AUC) = 1. The AUC can be used to distinguish a non-informative test (AUC = 0.5), a less accurate (0.5 < AUC ≤ 0.7), moderately accurate (0.7 < AUC ≤ 0.9), highly accurate (0.9 < AUC < 1), and perfect test (AUC = 1).

Within each MPI scoring system (MPI1, MPI2, and MPI3), a ROC curve AUC value was estimated for each analyte (pCO_2_, pO_2_, sodium, potassium, chloride, ionized calcium, glucose, osmolality, anion gap, and pH), and analytes with an AUC ≥ 0.7 were selected for inclusion in three final MPI scoring systems (MPI4, MPI5, and MPI6). For use of MPI4, total points for pH, pCO_2_, and potassium are selected from Table 1, and if the sum of the points is ≥10, turtles are categorized as physiologically deranged. Likewise, the MPI5 score is calculated using Table 2, including points for pH, pCO_2_, pO_2_, and potassium, with a cut-off score of ≥6; and the MPI6 score is calculated using Table 3, including points for pH, pO_2_, and potassium, with a cut-off score of ≥8. For example, using Table 1, a turtle with pH = 7.35 (6 points), pCO_2_ = 48 mmHg (6 points), sodium = 180 mmol/l (8 points), and potassium = 6.6 mmol/l (8 points) would receive a total MPI1 score of 28.

The sum of scores for these analytes was used to estimate SE, SP, positive predictive value (PPV), negative predictive value (NPV), positive likelihood ratio (LR+), and negative likelihood ratio (LR−) of each MPI at different cut-off values (Dohoo *et al.*, 2004). Given that the SE and SP of a MPI is a function of a cut-off value, ROC analysis was used to identify the MPI cut-off value where both sensitivity and specificity were optimized. The AUC values for MPI4, MPI5, and MPI6 were compared by a visual examination of the 95% confidence intervals for each MPI system. The commercial software MedCalc was used to perform the ROC analyses (MedCalc Statistical Software, v12.3.0; Mariakerke, Belgium). Finally, while SE and SP are fixed characteristics of a test, the PPV and NPV vary with the prevalence of disease (mortality). In order to simulate the diagnostic performance of MPI4, MPI5, and MPI6, the predictive values of positive and negative test results were estimated based on mortality proportions of 0.10–0.40.

## Results

Analysis of individual analytes in each initial MPI resulted in exclusion of several analytes with AUC <0.7 from each MPI (Tables 1, 2, and 3). Sodium was excluded from MPI1, leaving pH, pCO_2_, and potassium for this index, which is hereafter called MPI4. Glucose, sodium, and ionized calcium were eliminated from MPI2, leaving pH, pCO_2_, pO_2_, and potassium in this index (hereafter called MPI5). Glucose, sodium, ionized calcium, pCO_2_, anion gap, osmolality, chloride, and bicarbonate were eliminated from MPI3, leaving pH, pO_2_, and potassium in this index (hereafter called MPI6).

**Figure 1:**
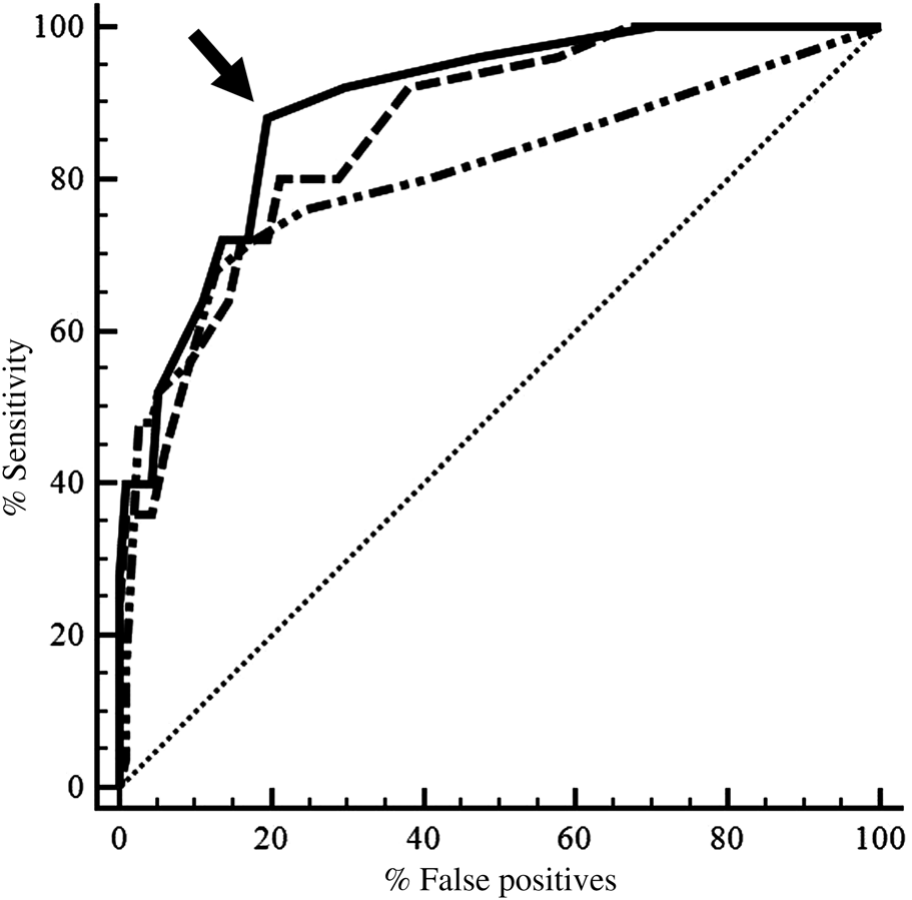
Diagnostic performance of mortality prediction indices MPI4 (dash-dotted line), MPI5 (dashed line), and MPI6 (continuous line) for identification of mortality in sea turtles (n = 25 dead and 118 live sea turtles). The diagonal (dotted) line indicates the area under the curve = 0.50 (a non-informative test). Arrow shows cut-off value of a score ≥8 for MPI6 with highest sensitivity = 88.0 and specificity = 80.5%.

Figure 1 shows a comparison of the diagnostic performance of MPI4, MPI5, and MPI6 by using ROC analysis. A visual observation of the three ROC curves revealed that the diagnostic performance of the three MPIs was similar. The AUC values for MPI4, MPI5, and MPI6 were 0.806, 0.864, and 0.896, respectively. The 95% confidence interval was 0.732–0.868 for MPI4, 0.797–0.916 for MPI5, and 0.834–0.941 for MPI6. Although the AUC for MPI6 (0.896) was the highest among the three MPI scoring systems, a visual examination of the 95% confidence interval further confirmed that the estimated AUC for each MPI was not different. Tables 4, 5, and 6 show the calculated SE, SP, PPV, NPV, LR + , and LR– values for MPI4, MPI5, and MPI6, respectively, at different cut-off values. Cut-off values that optimized AUC (e.g. SE and SP combined) were scores of ≥10, ≥6, and ≥8 for MPI4, MPI5, and MPI6, respectively. For use of MPI4, total points for pH, pCO_2_, and potassium are selected from Table 1, and, if the sum of the points is ≥10, turtles are categorized as physiologically deranged. Likewise, the MPI5 score is calculated using Table 2, including points for pH, pCO_2_, pO_2_, and K, with a cut-off score of ≥6; and the MPI6 score is calculated using Table 3, including points for pH, pO_2_, and potassium, with a cut-off score of ≥8.

**Table 4:** Sensitivity, specificity, predictive values and likelihood ratios of MPI4 using pH, pCO_2_ and potassium for identification of sea turtles that died or survived after admission to a rehabilitation centre

Cut-off point	Sensitivity	Specificity	PPV	NPV	LR +	LR −
4	80.00	59.32	29.40	93.30	1.97	0.34
6	76.00	75.42	39.60	93.70	3.09	0.32
8	72.00	82.20	46.20	93.30	4.05	0.34
**10**	**68.00**	**87.29**	**53.10**	**92.80**	**5.35**	**0.37**
12	56.00	90.68	56.00	90.70	6.01	0.49
14	52.00	94.92	68.40	90.30	10.23	0.51
16	48.00	95.76	70.60	89.70	11.33	0.54
18	48.00	97.46	80.00	89.80	18.88	0.53
20	32.00	98.31	80.00	87.20	18.88	0.69
22	16.00	99.15	80.00	84.80	18.88	0.85

Bold indicates the cut-off value ≥10 that produced the highest sensitivity and specificity combined. Abbreviations: LR + , positive likelihood ratio; LR − , negative likelihood ratio; NPV, negative predictive value; pCO_2_, partial pressure of carbon dioxide; pO_2_, partial pressure of oxygen; and PPV, positive predictive value.

**Table 5:** Sensitivity, specificity, predictive values and likelihood ratios of MPI5 using pH, pCO_2_, pO_2_, and potassium for identification of sea turtles that died or survived after admission to a rehabilitation centre

Cut-off point	Sensitivity	Specificity	PPV	NPV	LR +	LR −
3	96.00	42.37	26.10	98.00	1.67	0.09
4	92.00	61.86	33.80	97.30	2.41	0.13
5	80.00	71.19	37.00	94.40	2.78	0.28
**6**	**80.00**	**78.81**	**44.40**	**94.90**	**3.78**	**0.25**
7	72.00	80.51	43.90	93.10	3.69	0.35
8	72.00	83.90	48.60	93.40	4.47	0.33
9	64.00	85.59	48.50	91.80	4.44	0.42
10	56.00	90.68	56.00	90.70	6.01	0.49
11	44.00	94.07	61.10	88.80	7.42	0.60
12	36.00	95.76	64.30	87.60	8.50	0.67
13	36.00	97.46	75.00	87.80	14.16	0.66
14	36.00	99.15	90.00	88.00	42.48	0.65

Bold indicates the cut-off value ≥6 that produced the highest sensitivity and specificity combined. Abbreviations are as for Table 4.

**Table 6:** Sensitivity, specificity, predictive values and likelihood ratios of MPI6 using pH, pO_2_, and potassium for identification of sea turtles that died or survived after admission to a rehabilitation centre

Cut-off point	Sensitivity	Specificity	PPV	NPV	LR +	LR −
4	96.00	52.54	30.00	98.40	2.02	0.07
6	92.00	70.34	39.70	97.60	3.10	0.11
**8**	**88.00**	**80.51**	**48.90**	**96.90**	**4.51**	**0.15**
10	72.00	83.05	47.40	93.30	4.25	0.34
12	72.00	86.44	52.90	93.60	5.31	0.32
14	64.00	88.98	55.20	92.10	5.81	0.40

Bold indicates the cut-off value ≥8 that produced the highest sensitivity and specificity combined. Abbreviations are as for Table 4.

**Figure 2:**
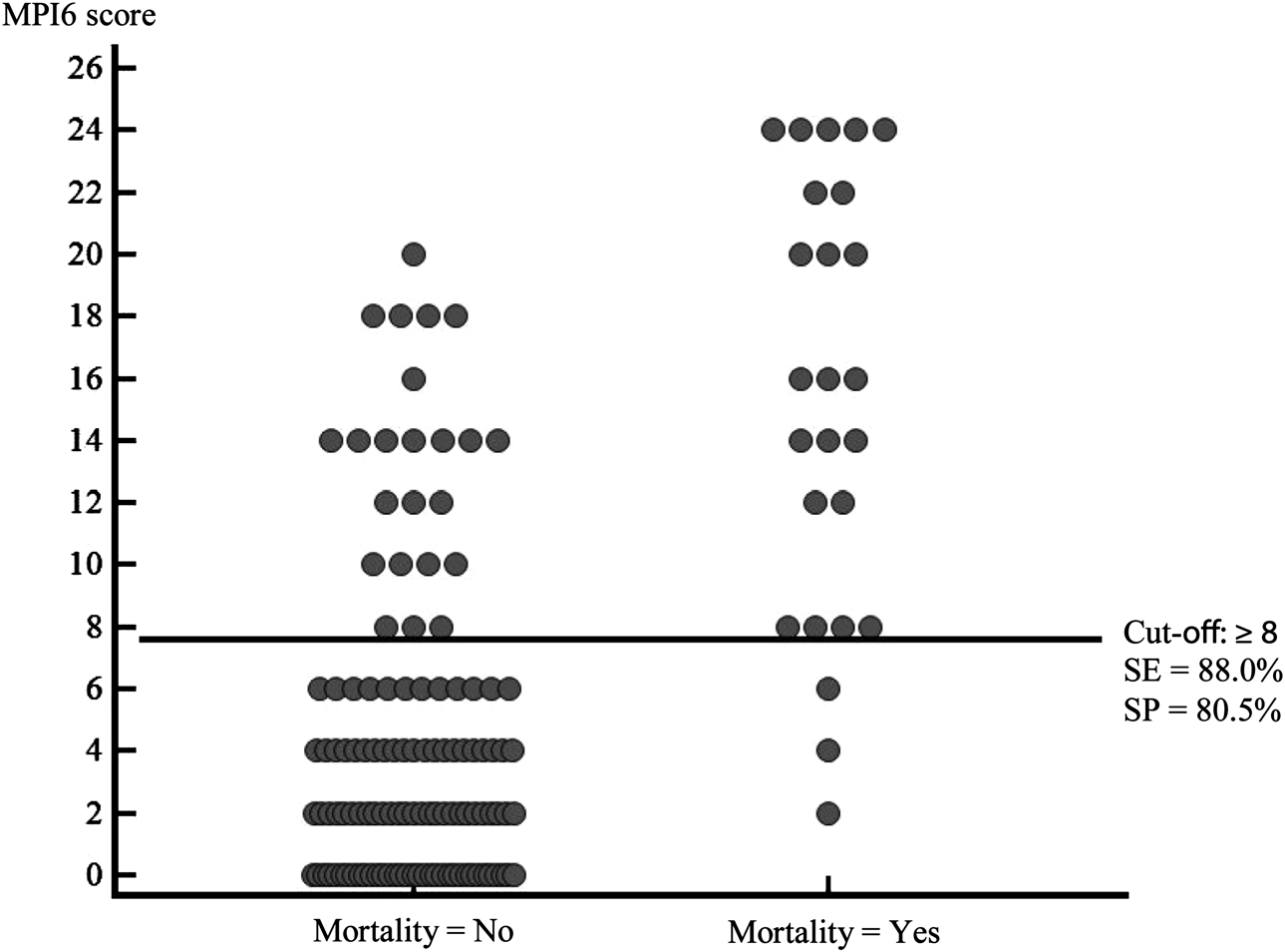
Frequency-distribution of MPI6 mortality prediction index scores in 118 sea turtles that survived and 25 sea turtles that died. The horizontal line across the MPI6 score indicates the cut-off value used to predict mortality (yes/no). Using a cut-off value ≥8, the calculated sensitivity = 88% and the specificity = 80.5%.

The MPI6 had the best combination of SE, SP, PPV, and NPV among the three MP indices. Using a cut-off value of ≥8, the SE and SP of the MPI6 were 88 and 80%, respectively (Fig. 1, Table 6); using the study mortality of 25 of 143 or 17.5%, the PPV and NPV were 48 and 96%, respectively. In addition, the LR + =4.51 and LR − =0.15 at the selected cut-off value. The study results also show that the specificity could be increased from 80 to 88% (by increasing the cut-off value from ≥8 to ≥14, thus increasing the PPV from 48 to 55%. The frequency-distribution of MPI6 scores in 118 sea turtles that survived and 25 sea turtles that died is presented in Fig. 2.

**Figure 3:**
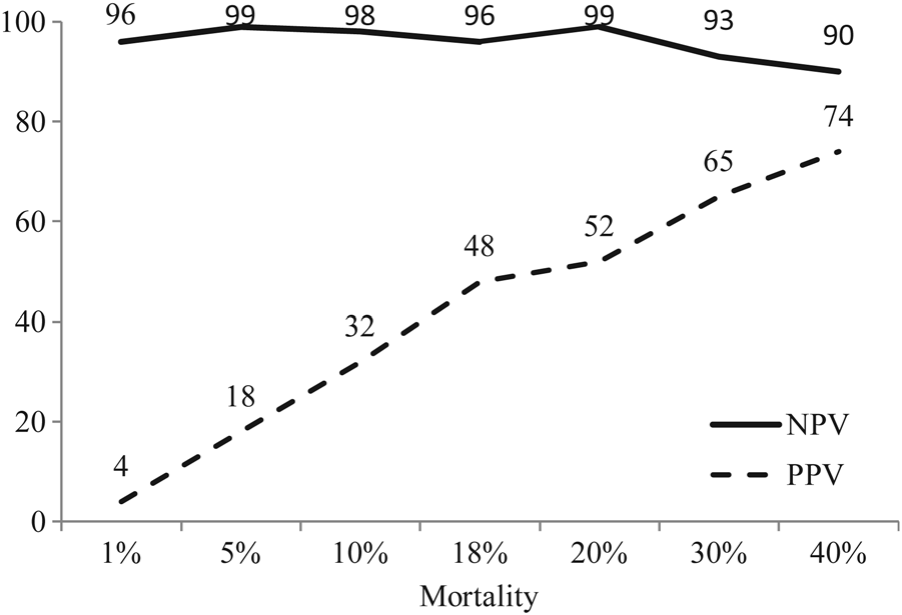
Relationship between mortality proportion, positive predictive value (PPV), and negative predictive value (NPV) for MPI6 (when the sensitivity = 88% and specificity = 80%).

Given that predictive values are affected by prevalence (i.e. mortality), Fig. 3 demonstrates the effect of mortality proportion on positive and negative predictive values of MPI6; the diagram shows a positive predictive value of ∼48% and a negative predictive value of ∼96% at the 17.5% mortality of the study population (Table 6); the positive predictive value will increase simultaneously with increased mortality.

## Discussion

Overall, using ROC analysis, all three final MPI scoring systems had an AUC >0.80; an indication that all three systems can be useful for predicting mortality in stranded, cold-stunned Kemp's ridley sea turtles. When using the MPI6 scoring system with a SE of 88%, a SP of 80%, a mortality of 17.5% observed in the study sample, and cut-off value ≥8, the NPV was very high, indicating that 96% of sea turtles with a score of 6 or lower were accurately identified as survivors during the first 7 days after admission to the NEAQ. The PPV (48%) suggests that half of sea turtles with a score ≥8 were accurately identified as turtles that died during the first 7 days after admission to the NEAQ. The study results also showed that increasing the SP from 80 to 88% would be of limited use, because the PPV would only increase from 48 to 55%. The PPV of a test can be influenced by the relative frequency of an illness event of interest. For example, in this study, if the mortality proportion had been higher (e.g. 0.40), the PPV of the MPI6 scoring system would increase to 74%, thus predicting mortality in three of every four sea turtles. Many cold-stunned sea turtles in the northeastern USA are not presented to rehabilitation facilities because they are presumed dead when found, or die during transport. Historically, 35–80% of cold-stunned turtles are already dead when found on the beach (Bentivegnal *et al.*, 2000; Gerle *et al.*, 2000; Turnbull *et al.*, 2000). The mortality for hospitalized turtles in this study (17.5%) is biased, because it excludes turtles that died prior to hospitalization, and is much lower than for turtles that do not receive medical care. Further bias is present to the extent that at least some turtles with very deranged biochemical results were probably treated more aggressively than turtles with normal results, such that some turtles with abnormal MPI scores survived when they otherwise would have died. The mortality in this study is similar to the historical mortality of 20% of hospitalized cold-stunned sea turtles in the northeastern USA (Wyneken *et al.*, 2006).

Although ROC analysis indicated that MPI6 had the highest AUC, it is important to note that MPI4 and MPI5 also performed very well, and may be useful for future investigations. It is notable that ROC analysis maintained pH and potassium concentration in all three models, pO_2_ in both models in which it was initially included, and pCO_2_ in two of three models. For MPI6, pCO_2_ was narrowly excluded, because its AUC was 0.696 (data not shown). Thus, consistent with previous reports, it is clear that pH, pCO_2_, pO_2_, and potassium concentration are of considerable relevance in the clinical assessment of cold-stunned Kemp's ridley turtles (Innis *et al.*, 2009b; Keller *et al.*, 2012). For example, cold-stunned Kemp's ridley sea turtles that died in one study were found to have higher initial blood pCO_2_ and potassium, and lower initial blood pH and pO_2_ than turtles that survived (Keller *et al.*, 2012). In the present study, the cut-off values applied to various abnormal ranges of the analytes for each MPI were developed based on the clinical experience of the authors (C.J.I., N.I.S.), and published data for each analyte relative to outcome. For example, we assigned highest scores to blood potassium concentrations >5.5 mmol/l, which have previously been associated with mortality in this species (Innis *et al.* 2009b; Keller *et al.*, 2012). For the majority of deaths in this study, derangements were almost always in the directions expected from previous studies, i.e. hypercarbia, hypoxia, acidosis, and hyperkalemia. For example, potassium derangements in turtles that died were almost always reflected by hyperkalemia, while marginally low potassium concentration (2.74 mmol/l) was seen in only one turtle that died. The good performance of the MPI scoring systems in this study suggests that the choice of analytes and point values assigned to various derangements were appropriate. The derangements seen in turtles in this study are consistent with those seen in studies of other physiological stressors, such as trawl net capture and forced submergence (e.g. Stabenau *et al.*, 1991; Harms *et al.*, 2003).

It is important to note that the pH, pCO_2_, and pO_2_ values used in this study were temperature-corrected using mathematical formulae that may be different from the formulae used by some blood analysers to perform automated temperature correction. While analyser auto-corrected values for pH and pCO_2_ of sea turtles have been shown to be clinically very similar to values calculated by the formulae used in this study, this is not true for pO_2_ results (Chittick *et al.*, 2002; Innis *et al.*, 2007). Thus, when using any of the MPIs described in this study that involve point assignments for specific pO_2_ values, it is important to use pO_2_ values that are temperature-corrected by the same formula used here, which may be quite different from pO_2_ values auto-corrected by the analyser.

Variability in sea turtle blood biochemical data has been reported between various analysers (Wolf *et al.*, 2008). The analyser used to generate the data evaluated in the present study has been validated, and is widely used in human and veterinary medicine (e.g. Flegar-Meštrić and Perkov, 2006; Acierno *et al.*, 2008). To our knowledge, it has not been validated for use with sea turtle blood by comparison to conventional bench-top equipment (e.g. radiometer), and has not been assessed in head-to-head comparisons with other analysers that have been evaluated in sea turtles. Such validation and assessments were beyond the scope of this study. Data generated by the analyser are clinically relevant in sea turtles, and clinically consistent with data produced for sea turtles using other methods (Innis *et al.*, 2007; Keller *et al.*, 2012). However, given the absence of species-specific validation, and lack of head-to-head comparison, results of this study should be applied cautiously to data that are generated by other analysers.

Assessment of the true utility of these MPI scoring systems will require application of the indices to future cohorts of Kemp's ridley and other sea turtle species, ideally from a variety of facilities, and under a variety of different stressors (e.g. trauma, fisheries interactions and intoxications). Some health scoring systems perform less well and require modification when applied to different facilities and cohorts of patients (King *et al.*, 2001). It is acknowledged that the MPIs in this study were applied to a cohort of juvenile cold-stunned Kemp's ridley sea turtles that was very similar (although involving different individuals) to those in previously published studies (Innis *et al.*, 2007, 2009b; Keller *et al.* 2012). As such, it was likely that the MPIs developed with knowledge of those previous studies could perform well in the present study.

The application of validated MPI scoring systems presented here could be of benefit in different clinical settings for improvement of diagnosis, treatment, and prognosis in cold-stunned sea turtles. For example, when managing large numbers of cold-stunned turtles during a large-scale stranding event, in which the mortality proportion is unknown at the time of admission, it may be reasonable for the MPI scores to be utilized to identify physiologically deranged animals in need of treatment. The MPI scores may also be useful in triaging the most debilitated turtles to facilities that can provide high-level critical care, while more stable turtles are sent to facilities with fewer resources or less experience. In addition, the MPI scores could allow for modification of appropriate therapy. For example, given the important influence of pH, pCO_2_, pO_2_, and potassium derangements on MPI, it is clear that clinical management of turtles with high MPI scores should focus on normalization of acid–base, blood gas, and electrolyte status via cardiorespiratory support and fluid therapy. At a mortality of 17.5%, the NPV of 96.9% of MPI6 implies that almost 100% of turtles with low scores will be likely to survive with appropriate treatment. This is clinically useful in making treatment decisions and in prioritizing available resources for turtles with the highest chance of survival. It is clear that results of MPI scoring systems cannot be used indiscriminately to make euthanasia decisions, because this would result in euthanasia of some turtles with a falsely positive MPI score that would otherwise survive. As with other health scoring systems in human and veterinary medicine, the MPI scores should not prevent clinicians from providing care to an individual, and euthanasia decisions should only be made in light of numerous other clinical factors, including neurological status, vision, ability to forage, ability to swim, pain and suffering, and duration of illness. Finally, MPI scores may be useful when applied retrospectively in a stranding event for comparison of various treatment outcomes within a facility or among different facilities. Thus, the MPI could provide an objective assessment tool of treatment success and contribute to the advancement of medical care in sea turtles.

## Funding

This work was funded by the National Oceanic and Atmospheric Administration.
